# Lipoxygenase 2 from *Cyanothece* sp. controls dioxygen insertion by steric shielding and substrate fixation

**DOI:** 10.1038/s41598-017-02153-w

**Published:** 2017-05-18

**Authors:** Julia Newie, Piotr Neumann, Martin Werner, Ricardo A. Mata, Ralf Ficner, Ivo Feussner

**Affiliations:** 1 University of Goettingen, Albrecht-von-Haller Institute for Plant Sciences, Department of Plant Biochemistry, Justus-von-Liebig-Weg 11, 37077 Goettingen, Germany; 2 University of Goettingen, Institute of Microbiology and Genetics, Department of Molecular Structural Biology, Justus-von-Liebig-Weg 11, 37077 Goettingen, Germany; 3 University of Goettingen, Institute for Physical Chemistry, Tammannstr. 6, 37077 Goettingen, Germany; 4 University of Goettingen, Goettingen Center for Molecular Biosciences (GZMB), Justus-von-Liebig-Weg 11, 37077 Goettingen, Germany

## Abstract

The biological function of lipoxygenases depends on the regio and stereo specific formation of fatty acid-derived hydroperoxides and different concepts exist to explain the mechanism that directs dioxygen to a specific carbon atom within the substrate. Here, we report the 1.8 Å resolution crystal structure of a cyanobacterial lipoxygenase that produces bis-allylic hydroperoxides (CspLOX2). Site directed mutagenesis experiments combined with computational approaches reveal that residues around the active site direct dioxygen to a preferred carbon atom and stereo configuration in the substrate fatty acid. Modulating the cavity volume around the pentadiene system of linoleic acid shifted the product formation towards 9*S*-, 9*R*-, 13*S*- or 13*R*-hydroperoxides in correlation with the site of mutation, thus decreasing the amount of the bis-allylic 11*R*-hydroperoxide. Decreasing the channel size of a 9*R*-lipoxygenase (CspLOX1) on the other hand could in turn induce formation of the bis-allylic 11*R*-hydroperoxide. Together this study suggests that an active site clamp fixing the pentadiene system of the substrate together with steric shielding controls the stereo and regio specific positioning of dioxygen at all positions of the reacting pentadiene system of substrate fatty acids.

## Introduction

Lipoxygenases (LOX) are key enzymes in the initial step of lipid-mediator biosynthesis. They catalyze a regio- and stereo specific dioxygenation of polyunsaturated fatty acids to yield fatty acid hydroperoxides, which can be further metabolized to a variety of signaling molecules with diverse biological functions^1, 2^. LOX are ubiquitous in mammals and plants, but have also been found in some fungi and prokaryotes^3^.

The LOX reaction is always catalyzed at a 1*Z*,4*Z*-pentadiene system of a polyunsaturated fatty acid. In the initial step, a hydrogen atom is abstracted from the central bis-allylic methylene group by the non-heme iron or in few cases manganese in the active site^4^. During this rate-limiting step, Fe(III) is reduced to Fe(II) in the former case and a substrate radical is formed which rapidly delocalizes over the five carbon unit. Addition of dioxygen yields a peroxyl radical, which is finally reduced to the hydroperoxide product^5^. Dioxygen is usually directed with high regio and stereo selectivity to the n + 2 or n − 2 position relative to the carbon atom “n” at which the hydrogen has been abstracted. This selectivity has a great impact on the biological function of the products. In mammals, for example, the arachidonate 5-LOX activity is the key reaction for the production of pro-inflammatory leukotrienes while a combination of 5-LOX and leukocyte 12/15-LOX activity is associated with the production of anti-inflammatory lipoxins^6, 7^. In plants, the positional specificity is of similar importance, as only (13*S*)-hydroperoxy octadecatrienoic acid (13-HPOTE) but not 9-HPOTE is the precursor for the plant hormone jasmonic acid that is crucial for defense responses and regulation of developmental processes^8^.

In addition to these classical LOX, which produce only hydroperoxide products with a conjugated double system, few LOX catalyze the hydroperoxide formation at the bis-allylic carbon atom, which corresponds to C11 of linoleic acid (18:2n-6). These LOX are mainly fungal enzymes that contain manganese in the active site^9–11^, but also the iron-containing enzyme CspLOX2 from the cyanobacterium *Cyanothece* PCC8801 forms (11*R*)-hydroperoxy octadecadienoic acid (11-HPODE) as major product^12, 13^. Even though the first enzyme with bis-allylic products was reported in 1998^9^ and the first structures of a manganese-containing LOX were recently published^14, 15^, the underlying structural factors responsible for this unusual reaction could not yet be determined.

In contrast to these bis-allylic LOX, the relationship of structure and dioxygenation specificity has been studied for classical LOX that produce only conjugated products^4^. Here, the basis is the positioning of the reacting pentadiene relative to the iron. Firstly, the depth of the substrate-binding pocket determines how deep the fatty acid substrate can slide into the active site to expose a certain pentadiene to the catalytic iron^16, 17^. Secondly, it has been shown that the substrate binding mode is important, as different faces of the fatty acid are oriented towards the iron depending on the forward or head-to-tail binding mode^18^. Thirdly, the access of molecular dioxygen is regulated by a glycine/alanine-positional switch steering the dioxygen positioning between the two terminal positions on the pentadiene system^19^.

On the basis of the CspLOX2 crystal structure obtained in this study, we analyzed hypothetical structural factors forming the environment of the pentadiene system that may explain the unusual specificity. We demonstrate the importance of an active site clamp around the pentadiene system, which fixes the pentadiene during the reaction resulting in 11-HPODE formation. Furthermore, we propose that several key residues in the active site efficiently shield C9 and C13 of linoleic acid from dioxygen thus leaving only C11 accessible for dioxygen insertion.

## Results

### CspLOX2 structure resembles the C-terminal domain of prototypical LOX

**Figure 1:**
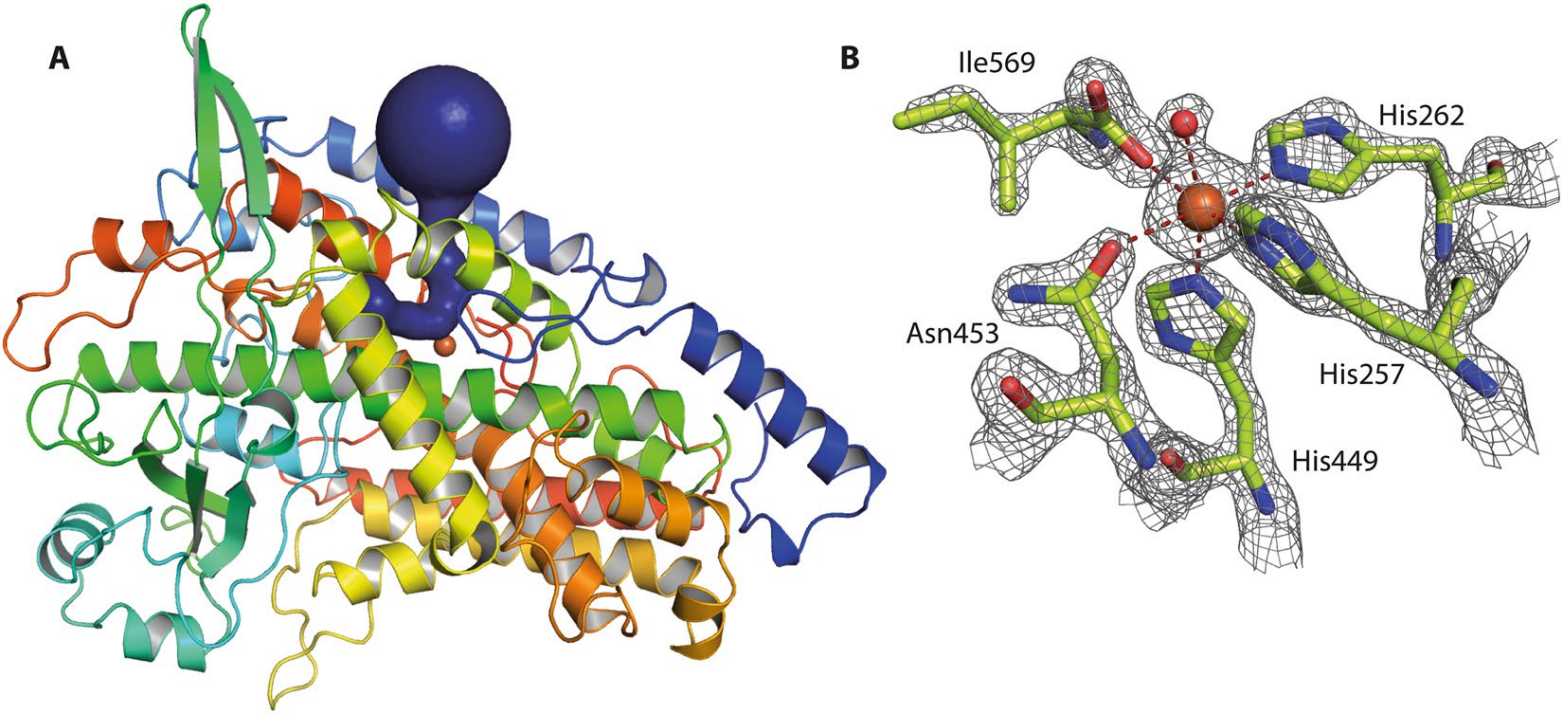
Structure of CspLOX2. (**A**) The overall structure of CspLOX2 is depicted with its putative substrate-binding channel calculated by Caver 3.0 shown in blue and the catalytic iron as orange sphere. (**B**) The catalytic iron is coordinated by five amino acids and a water/hydroxide in an octahedral geometry. The *2mFo-DFc* electron density map is contoured at 4 σ. The red dashed lines highlight coordination geometry of the metal ion.

CspLOX2 is the LOX with the highest relative amount of bis-allylic product reported so far^13^, hence it is a suitable candidate to study the molecular basis of the dioxygen positioning at the bis-allylic position. We therefore crystallized the enzyme and determined its structure to 1.8 Å resolution (PDB code 5MED). CspLOX2 crystallized in space group *P*2_1_2_1_2_1_ with one homodimer in the asymmetric unit. The overall structure of CspLOX2 resembles the C-terminal catalytic domain of other LOX and consists mainly of α-helices (root-mean-square deviation [RMSD] of 1.59 Å for 514 common Cα-atoms with coral 8*R*-LOX [PDB code 4QWT]). As revealed by sequence alignments of CspLOX2 with other LOX^12^, the N-terminal C2-like domain, which is a common feature of eukaryotic LOX, is absent in CspLOX2 (Fig. 1A). A well-defined boot-shaped channel connects the surface of the protein with its active site. This channel is lined by hydrophobic amino acids and is most likely involved in the positioning of the fatty acid substrate. The catalytic non-heme iron, deeply buried in the CspLOX2 active site, is coordinated by three invariant histidines (His257, His262, His449), Asn453 and the carboxy group of the C-terminal Ile569 (Fig. 1B). The sixth ligand of the octahedrally coordinated iron, which is positioned towards the putative substrate-binding channel, is a water (Fe^2+^-H_2_O) or hydroxide molecule (Fe^3+^-OH) acting as a catalytic base for hydrogen abstraction during catalysis. In contrast to MnLOX, for which a slightly altered metal coordination geometry was reported^14, 15^, the coordination of the iron cofactor in CspLOX2 resembles strongly the one of the prototypical soybean LOX1 or the 8*R*-LOX from *P. homomalla* (Supplemental Fig. 1). Interestingly, even when the iron cofactor is replaced by manganese^13^, the coordination geometry is not affected (PDB code 5MEG, Supplemental Fig. 2.

### Residues of the active site clamp control the regio and stereo specificity

**Figure 2:**
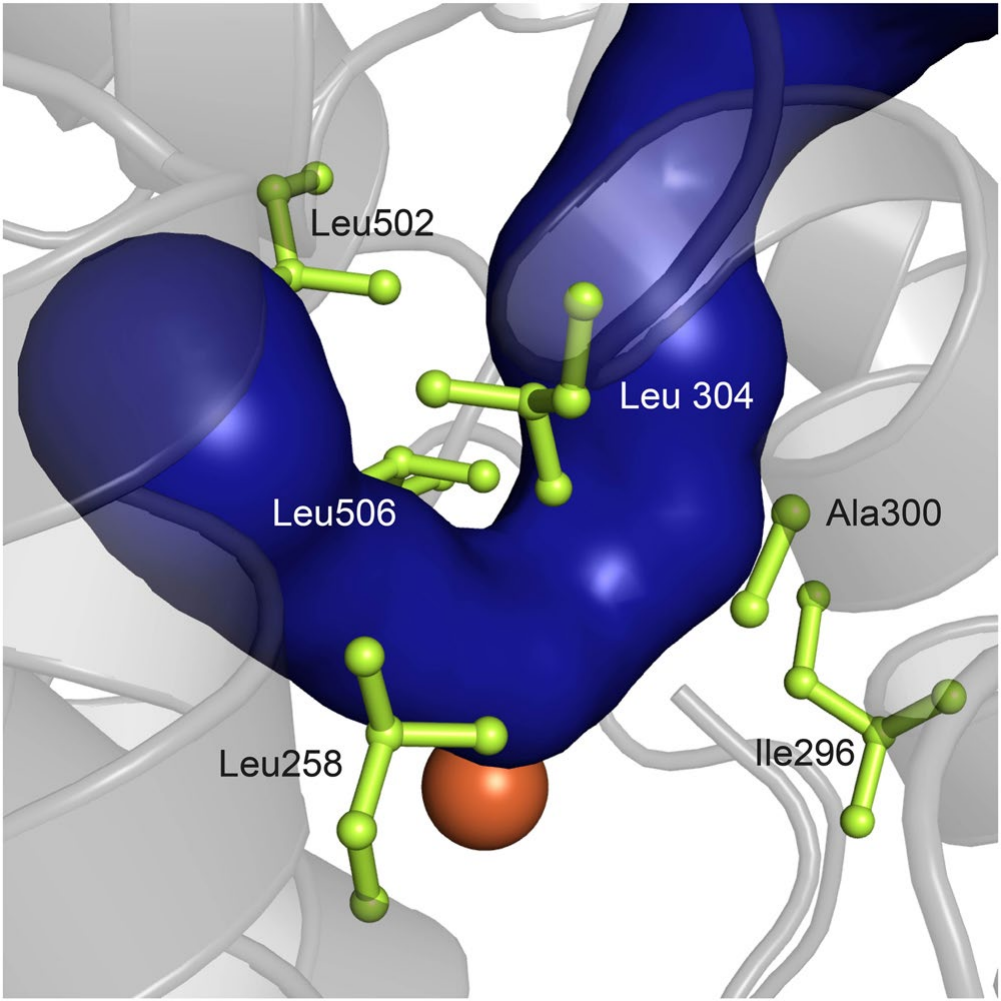
Residues forming a clamp-like structure around the CspLOX2 active site. The putative substrate-binding channel is shown in blue, the catalytic iron as orange sphere and the side chains of the crucial active site amino acids in light green.

**Figure 3:**
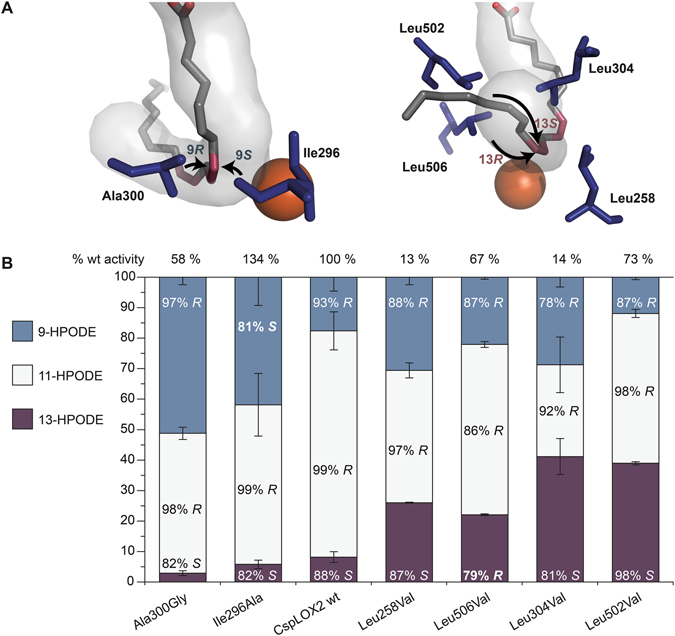
Mutations of the active site clamp affect the regio and stereo specificity of CspLOX2. (**A**) The conformation of the arachidonic acid (20:4n-6) substrate crystallized in the coral 8*R*-LOX (grey sticks) fits quite well into the predicted channel of CspLOX2, which is shown from the entry part (left) and from the bottom part (right). The double bonds that belong to the reacting pentadiene are shown in dark red. The residues shown in blue belong to the active site clamp with Ala300 and Ile296 closer to the n-2 which corresponds to position C9 on linoleic acid (18:2n-6) and with Leu258, Leu304, Leu502 and Leu506 closer to the n-2 position (C13 on linoleic acid). (**B**) The residues shown in (**A**) were exchanged by smaller residues and the product distribution was analyzed by HPLC. Shown are mean values and error bars show standard deviation. Additionally to the distribution of the positional isomers 9-HPODE, 11-HPODE and 13-HPODE, the chirality of each product is indicated. The relative activities shown on top were determined by measuring the consumption of oxygen. All experiments were carried out in triplicates.

As the bis-allylic 11-HPODE product may be formed either by manganese or iron LOX^12, 13, 15^, we searched for structural features in the amino acid environment that might act as determinants for 11-HPODE production. Despite extensive efforts, we could not obtain crystals of a CspLOX2 substrate complex. However, the structure of a coral 8*R*-LOX with bound substrate (arachidonic acid, 20:4n-6) was recently published (PDB code 4QWT) and provided insights into the substrate binding in the active site^20^. In order to learn more about the substrate binding in CspLOX2, we focused on the conformation of the second pentadiene system of arachidonic acid that comprises of the double bond positions Δ8 and Δ11, which fit best to the double bond positions Δ9 and Δ12 in linoleic acid as an approximation. Both structures were superimposed and the possible location of the fatty acid in the active site channel of CspLOX2 was analyzed. Using this structural model, we generated different CspLOX2 variants with single amino acid substitutions altering this channel. In general, mutations inserted close to the iron in the kink of the channel had the strongest effects on the product specificity, while amino acid substitutions closer to the entrance or the bottom of the channel resulted in smaller changes (Supplemental Fig. 3). A key residue that is located opposite of the iron ion and hence likely restricts the space in the kink region is the highly conserved Leu304 (Figs 2 and 3A). Exchange of this residue for a less bulky valine reduced the relative amount of 11-HPODE to 30% compared to 74% produced by CspLOX2 wt (wild type) (Fig. 3B). The crystal structure of the Leu304Val variant (PDB-code 5MEE) revealed that the active site channel was indeed widened in the kink region by the mutation, while having virtually no effect on the rest of the protein structure (Supplemental Fig. 4). This may suggest that the restricted space in the kink region is required for effective 11-HPODE formation by CspLOX2, as a tight and kinked substrate-binding channel might induce slight distorsions in the reacting pentadiene system, thus altering the reactivity. This possibility was addressed by a theoretical approach (see below). Exchanging this key residue for an even smaller residue (Leu304Ala) could, however, not further decrease the relative amount of 11-HPODE formation, nor could bulkier residues (Leu304Phe, PDB code 5MEF) increase it (Supplemental Figs 3 and 4C).

Besides Leu304, we could identify further crucial residues in the direct environment of the narrow active site that form a clamp-like structure, including Leu258, Ile296, Ala300, Leu502 and Leu506 (Fig. 2). Of these residues, leucines were exchanged for valines, alanine for glycine and isoleucine for alanine, respectively, thus allowing more space at different parts of the active site. All these variants, which retain at least 13% of wt activity, produce a slightly reduced amount of 11*R*-HPODE compared to wt CspLOX2 (Fig. 3). But more surprisingly, mutations at these residues resulted in remarkable changes in product composition, so that amounts of all of the possible conjugated HPODE from linoleic acid, namely 9*S*-, 9*R*-, 13*S*- and 13*R*-HPODE, could be increased substantially, depending on which amino acid was decreased in size. More precisely, mutations closer to the entrance of the channel resulted in high amounts of 9-HPODE, with 9*R*-HPODE produced by the Ala300Gly variant and 9*S*-HPODE produced by the Ile296Ala variant (Fig. 3). The mutations closer to the bottom of the channel, Leu506Val and Leu502Val, resulted in high amounts of 13*R*-HPODE and 13*S*-HPODE, respectively. Compared to wt CspLOX2, the enantio selectivity towards formation of 9*R*- and 13*S*-HPODE was even increased by Ala300Gly and Leu502Val, respectively, while the stereo specificity was inverted by Ile296Ala and Leu506Val, respectively. In contrast to this, the effect of Leu258Val was intermediate, with only a slight shift towards 13*S*-HPODE. This is to our knowledge the first study in which the product specificity of a single LOX could be substantially redirected towards either 9*S*, 9*R*, 13*S* or 13*R*-HPODE by single amino acid substitutions in the active site. Of the identified residues, Ala300 is already known as key player (Coffa-Brash determinant) for directing dioxygen to a specific side on the substrate, with *R*-specific LOX having a glycine and *S*-specific LOX having an alanine at this position^19^. In line with the proposed model^21^, the exchange to the alternative glycine residue resulted in a further increase of 9*R*-HPODE over the other products (Fig. 3B). The other residues of the identified active site clamp have not been studied to this extend before.

The sites of the substrate in our structural model where dioxygen may be added to linoleic acid to form 9*R*- and 9*S*-HPODE pointed to the positions of Ala300 and Ile296, respectively (Fig. 3A). In case of 13-HPODE, Leu304 and Leu502 are rather facing the side of 13*S*-HPODE formation, while Leu506 faces the side for 13*R*-HPODE formation. Consequently, if more space is created by decreasing the size of one amino acid, dioxygenation at the nearest position on the linoleic acid substrate is preferred over other possible dioxygenation sites. These results suggest that steric shielding of the substrate controls the formation of 11*R*-HPODE by CspLOX2 and that not only the orientation and depth of substrate binding, but also the direct environment of the pentadiene system plays a crucial role for the positional and enantioselective specificity of LOX.

### CspLOX2 specific residues induce 11-HPODE formation in CspLOX1

**Figure 4:**
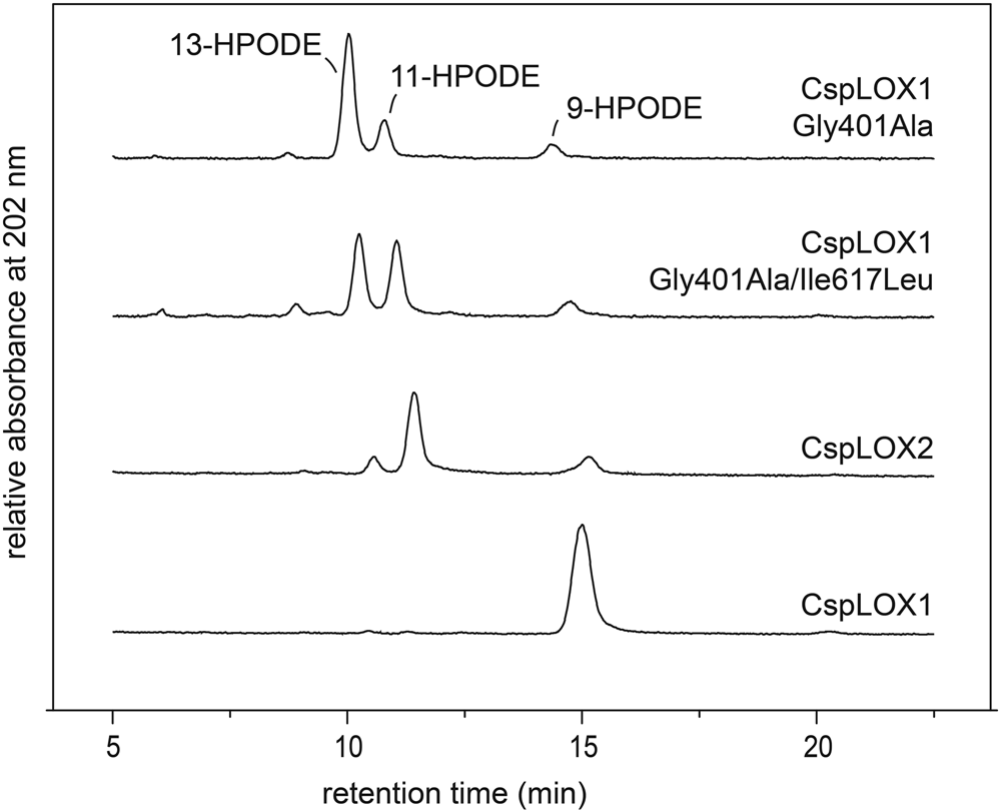
Two amino acid substitutions in CspLOX1 induce 11-HPODE production. Chromatogram of the HPLC analyses of products formed by the Gly401Ala and the Gly401Ala/Ile617Leu variant of CspLOX1 as well as the wt of CspLOX2 and CspLOX1. While CspLOX1 only produces 9-HPODE, the Gly401Ala variant and the Gly401Ala/Ile617Leu variant produce significant amounts of the bis-allylic 11-HPODE that is characteristic for CspLOX2.

In a gain of function approach, we wanted to further elucidate whether steric shielding plays indeed a role in 11-HPODE formation. We hence aimed at modifying the active site of another LOX that only produces conjugated HPODE in a way that it imitates CspLOX2 and forms 11-HPODE. The second characterized LOX from *Cyanothece* PCC8801, CspLOX1, seemed to be a suitable enzyme for this experiment since it originates from the same organism, a crystal structure is available and it produces no 11-HPODE^22^. However, CspLOX1 shows only 19% sequence identity with CspLOX2. Focusing on the active site clamp, we found that three residues are invariant: Ile379, Leu405 and Leu621, which correspond to Ile296, Leu304 and Leu506 in CspLOX2. However, three of the active site residues, Tyr360, Gly401 and Ile617, differ in CspLOX1 and were therefore replaced by respective residues from CspLOX2 (Leu258, Ala300 and Leu502) (Table 1). Besides single amino acid exchanges, the mutations were also combined to yield double and triple mutants. Although these mutations resulted in a decrease in enzymatic activity (Supplemental Fig. 5), especially when multiple mutations were introduced, two variants, which retain 5-8% of wt activity, indeed produced 11-HPODE. The Gly401Ala variant produced 6% 11-HPODE, which was increased to 11% in combination with Ile617Leu (Fig. 4). The identity of this product was confirmed by mass spectrometry (Supplemental Fig. 6). Interestingly, the exchange of Ile617Leu and Gly401Ala may restrict the space in the active site close to C9 and C13 of linoleic acid. These data further support the hypothesis that residues of the active site clamp are important for the enzyme’s ability to produce 11-HPODE by restricting the space around the pentadiene system.

**Table 1:** Residues corresponding to the active site clamp in other LOX.

		**9** S*	**9** R*		**13** S*	**13** R*	Main product
CspLOX2	**Leu258**	**Ile296**	**Ala300**	**Leu304**	**Leu502**	**Leu506**	**11** ***R***
CspLOX1	Tyr360	Ile397	Gly401	Leu405	Ile617	Leu621	9*R*
coral 8*R*-LOX	Leu758	Ile796	Gly800	Leu804	Thr996	Leu1000	8*R*
human 15*S*-LOX	Leu361	Ile399	Ala403	Leu407	Ile592	Leu596	15*S*
*P. aeruginosa* LOX2	Leu378	Ile416	Ala420	Leu424	Ile608	Leu612	15*S*
13*R*-Mn-LOX (*G. graminis*)	Val291	Ile328	Gly332	Leu336	Leu535	Phe539	13*R*
9*R*-Mn-LOX (*M. salvinii*)	Val299	Val337	Gly341	Leu345	Leu536	Phe540	9*R*
soybean LOX-1	Trp500	Ile538	Ala542	Leu546	Val750	Leu754	13*S*

While most of these residues are rather conserved among LOX, Leu502 and Leu258 show a higher variability. The specificities observed by exchange of these residues in CspLOX2 are shown with an asterisk. these residues in CspLOX2 are shown with an asterisk.

### DFT calculations on spin localization and activation barriers

In order to better understand the results obtained by the mutational studies and to elucidate the mechanism behind the dioxygen positioning specificity, we carried out a series of theoretical calculations. They included electronic structure and molecular mechanics computations and were devised to test the several proposed factors steering the specificity, namely: spin localization, substrate conformation, as well as sterical constraints in the active site. The existence of an oxygen channel was not directly considered but will be discussed in the context of our model calculations.

**Figure 5:**
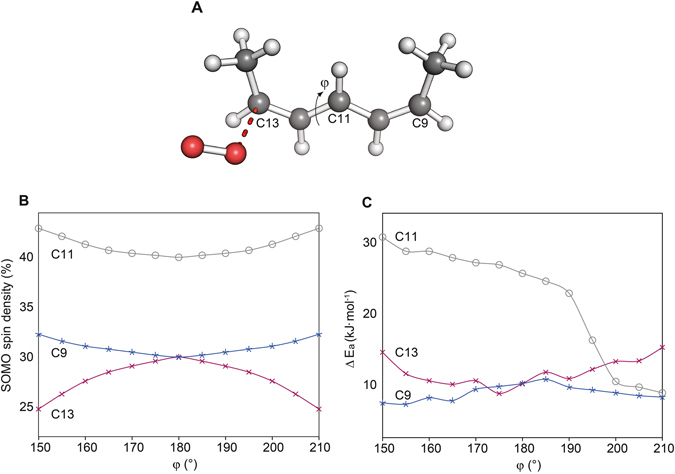
Model calculations exhibit different expectations for substrate spin density and activation barriers of oxygen attack. (**A**) Structure of the 2,5-heptadienyl radical which was used as a model system for the substrate. The correspondence to the carbon atoms of linoleic acid (18:2n-6), the varied dihedral angle φ and the side for oxygen attack are shown. (**B**) Spin density of the singly occupied molecular orbital (SOMO) on the three carbon atoms of interest for different dihedral angles φ. (**C**) Activation barriers for oxygen attack at the three carbon atoms of interest for different dihedral angles φ obtained by relaxed surface scans.

As it was pointed out before a simple explanation for the observed selectivities would be, that the enzyme induces slight out-of-plane bending within the substrate leading to a spin localization at specific carbon atoms and thus making these sites more reactive for molecular dioxygen^23, 24^. To test this, we performed density functional theory (DFT) calculations on a 2,5-heptadienyl radical (Fig. 5A) modeling the linoleic acid radical bound to the active site of CspLOX2. The amount of spin density of the singly occupied molecular orbital (SOMO) on the carbon atoms corresponding to C9, C11 and C13 was calculated varying an internal dihedral angle from 150° to 210° in steps of 5° (Fig. 5B). As expected, the spin density is higher at the central C11 position than it is at the outer C9 and C13 carbons for the preferred planar substrate conformation. By slight twist of the C13 carbon out of the substrate plane spin localization can be induced as the overlap of the conjugated π-orbitals becomes less effective. This leads to a reduced amount of spin density found at C13 while spin localization is increased at C9 and C11. However, substrate out-of-plane twisting is not sufficient to vary the carbon site carrying the highest amount of spin density as the fraction of SOMO spin density is larger at C11 than it is at C9 and C13 for all conformations. If the favored position for dioxygen addition was only controlled by the spin density distribution, no change in the main product of the enzyme could be obtained. Thus, spin localization does not provide a suitable explanation for the different selectivities observed.

In order to further analyze the competing reactions, we calculated activation barriers of dioxygen attack at the three different carbon atoms of interest by approaching molecular oxygen in relaxed surface scans with a varying C10-C11-C12-C13 dihedral angle (Fig. 5C). In contrast to the expectations based on the spin density analysis, C11 exhibits a significantly higher activation barrier than C9 and C13 considering the preferred planar substrate structure. This follows the general trend for product formation^25^, but falls in disagreement with the calculations of Hu and Pratt^26^. The latter found that the barrier for insertion of O_2_ in a pentadienyl radical to the central carbon atom would be about 1 kcal/mol higher in energy than to an end position. There are several reasons behind this mismatch. First of all, the model system is different, as we make use of a 2,5-heptadienyl model, adding two extra methyl groups to the ends to mimic the extended alkane chain neighboring the double bonds. Although a small change, this has a significant impact in the barriers. While in the case of the pentadienyl radical two rather different barriers are found for *syn* and *anti* addition, in our model there is little to no difference between the two. The *anti* addition barrier is much lower and both values agree well with the aforementioned study (around 12 kJ/mol). The only remaining difference is in the barrier for the central addition. Our reaction paths have been optimized with the C11-C12 and C10-C11 dihedrals fixed. As one can see in Fig. 5, a rotation of about 20° lowers the barrier for C11 addition significantly. In the unconstrained energy path for central addition, Hu and Pratt obtained a transition state with one of the dihedrals rotated by about 13°. Such an angle, according to Fig. 5, would be enough to bring the barrier height down to 17 kJ/mol, in good agreement with their DFT computed value.

Given the simplicity of the model, we cannot provide a full quantitative prediction of the different barrier heights in the real system. We can, however, conclude that an imposed torsion of the substrate could benefit the C11 addition. On the other hand, it would do little to change the preference towards C9 or C13.

### Molecular dynamics simulations support a major role of steric shielding of active site clamp

For the analysis of substrate conformation and environment we placed the enzyme with a bound linoleic acid radical substrate in a water box and performed molecular dynamics (MD) simulations for the wt CspLOX2 and its single amino acid mutants. Our intention was to find specific substrate conformations with well-defined dihedral angle distributions for each of these mutants in order to correlate them with the activation barriers of the model system. However, comparable dihedral angle distributions with a maximum close to the preferred planar substrate structure were obtained for all of these cases (Supplemental Fig. 7). The observed selectivities, therefore, should not be a result of enzyme-induced substrate torsions. Similar conclusions were drawn by Furse *et al*.^23^, who observed that the substrate conformation was mostly kept from solution to the active site for a cyclooxygenase system. Having ruled out spin localization and the torsional conformation as major determining factors for the regio selectivity, we turn to alternative hypothesis. The most likely explanation would be a control through steric shielding of the enzyme environment, either through changes in a tentative oxygen channel or by blocking access to the different carbons. The former possibility was already recognized as part of separate oxygen channeling^27^. In order to investigate to investigate this in further detail, we employed a theoretical model for steric shielding. Conventionally, the latter is analyzed by simply considering distances to the reactive site (may this be an atom or moiety). However, this is flawed when considering the current reaction since O_2_ must approach the carbons in an appropriate orientation (orbital overlap must be considered). The van der Waals minimum is the position, which should be considered, not the carbons in the substrate.

**Figure 6:**
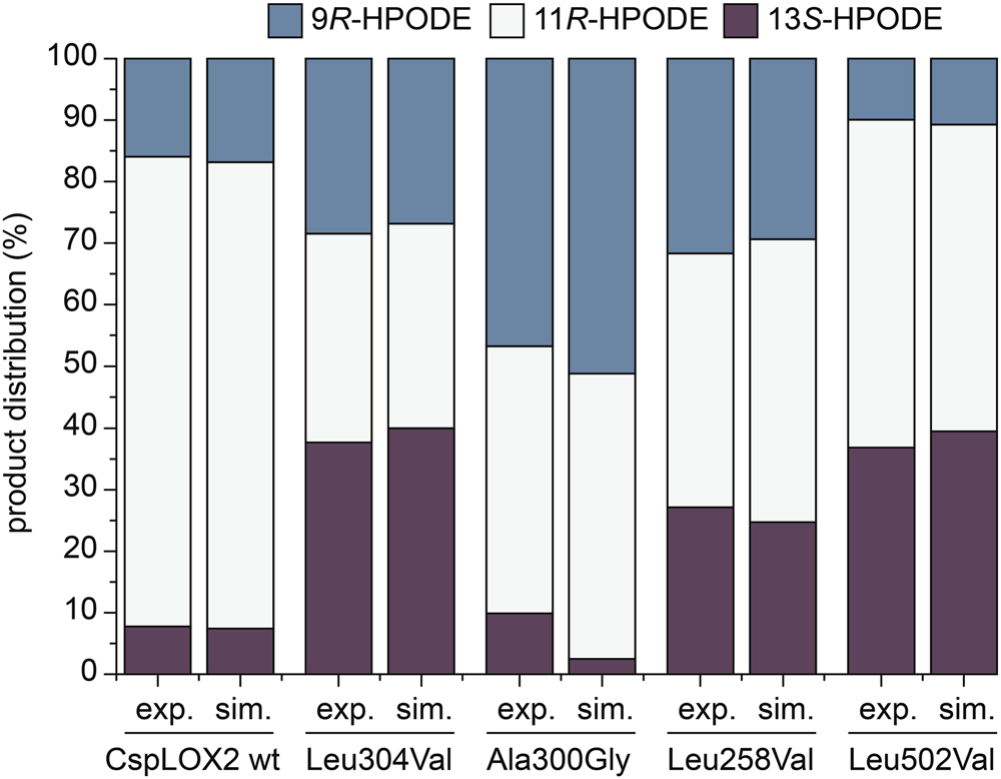
Product distributions for an antarafacial oxygen attack in CspLOX2 and its mutations. The distributions of the main stereo products of the CspLOX2 wt and a set of single amino acid mutations were calculated from MD simulations using a steric shielding model. The amount of C11 product was lowered by 10% and equally distributed to C9 and C13 in order to take the higher activation barrier of an oxygen attack at C11 into account. These results (sim.) are compared to the experimental distributions for these products (exp.) shown in Fig. 3.

We took as a constraint that dioxygen will always be added to the side opposite to the iron. Given that this corresponds exclusively to 9*R*, 11*R* and 13*S* products, this model is restricted to the study of mutants with the same stereo specificity as the wt protein. We carried out MD simulations for all these mutants at ambient temperature with production times of 1 ns (more information about the setup and the runs can be found under Materials and Methods in the Computational Details section). For each snapshot of every MD simulation an equalization plane was defined through the pentadiene unit of the substrate. By using the normal of this plane a point of 3 Å distance to the carbon atom on the antarafacial side of the substrate was found for C9, C11 and C13 respectively. This distance corresponds to the van der Waals minimum found in our initial reaction path calculations. If there was no atom found within a sphere of 2.2 Å around the defined point the corresponding carbon site was considered to be fully accessible as the space is sufficient to form the pre-reactive complex needed for oxygen addition. If there was an atom found closer than 1.8 Å to the defined point the carbon site was counted as completely shielded as the space is insufficient to allow molecular oxygen to approach. For intermediate distances from 2.2 Å to 1.8 Å an exponential damping function was used in order to account for flexible movements during the simulations without complete change of the carbon site accessibility (Supplemental Fig. 8). With this model for steric shielding a distribution of the products of antarafacial oxygen attack (9*R*, 11*R* and 13*S*) was obtained for each MD simulation by using the distribution of accessible carbon sites. A penalty was applied reducing the amount of C11 product by 10% and distributing them equally to C9 and C13 in order to respect the higher activation barrier for oxygen attack at this position. For each enzyme conserving the stereo products of the wt the calculated product distributions of three different MD simulations were averaged and compared to the experimental distribution. Results are shown in Fig. 6. The agreement between the computed and the experimental results is remarkable. All calculated distributions reproduce the experimental selectivities with a maximum deviation (MAXD) of 7.4%. The RMSD over all products of the considered enzymes is only 3.1%. These results appear to confirm that steric shielding is the main factor of selectivity control. It should be noted that this is the first theoretical model to accurately replicate the specificity of several LOX mutants.

Still unexplained is the change in enantioselectivity for the Ile296Ala and Leu506Val mutants, which we did not consider in our model. Our calculations were carried out under the constraints that the oxygen addition always occurs on one side of the substrate. Considering the success of our model for the CspLOX2 wt and all enantioselectivity conserving mutants, it seems unlikely that such a change would arise due to an oxygen channel switching sides. More likely, the substrate in the lle296Ala and Leu506Val mutants will be constrained in such a way that the affected carbon site is flipped. We were, however, unable to reliably probe such a change in the conformation. This is a subject for ongoing calculations.

## Discussion

This study demonstrates the importance of the active site clamp around the pentadiene for the formation of the bis-allylic product. Our results strongly suggest that the surrounding amino acids, which fix and shield the pentadiene system of the substrate during the reaction, play a major role in 11-HPODE formation. Although the formation of hydroperoxide product at the bis-allylic carbon is an unusual phenomenon, it is important to understand how this reaction is catalyzed. In autoxidation, molecular oxygen reacts with a linoleate carbon radical at C9, 11, or 13, although on account of the rapid on-off transitions of the resulting peroxyl radical at C-11, the 11-hydroperoxide is not formed without the addition of an efficient radical trap^28^. Our calculations on the 2,5-heptadiene radical model system suggest that reaction of O2 is slightly disfavored at the bis-allylic position (Fig. 5), therefore the enzyme needs to apply both steric control and efficient radical trapping to complete this thermodynamically unfavorable reaction. By elucidating how dioxygen is specifically inserted at the middle position of the pentadiene, we may also learn how it is directed to any other position on the pentadiene system. The bis-allylic product has so far only been reported for a few LOX. Most of these are fungal LOX containing manganese instead of iron in the active site^9–11^. Since CspLOX2 harbors iron in the active site and is inactive with manganese, the determinant for this unusual reaction cannot be, at least for CspLOX2, the type of metal in the active site^13^. The recently published structures of MnLOX revealed that the geometry of manganese coordination in the active site differs from the iron coordination in FeLOX^14, 15^. The coordination geometry of CspLOX2, however, resembles those of other FeLOX (Supplemental Fig. 1A) even if manganese was incorporated into CspLOX2 (Supplemental Fig. 2). Although the altered coordination geometry of MnLOX is most likely not connected to 11-HPODE formation, it might be necessary to complete the catalytic cycle which was not possible with manganese-substituted CspLOX2^13^.

In this study we particularly focused on residues around the active site, since dioxygen is added directly at the carbon atom at which hydrogen was initially abstracted. We found that the ability to produce 11-HPODE was diminished by widening the active site clamp in CspLOX2 (Fig. 3), while the production of 11-HPODE could be induced in its isoenzyme CspLOX1 by reducing the space in the channel (Fig. 4). Consequently, a tight channel close to the iron might be connected with the synthesis of 11-HPODE and this is supported by the structures of MnLOX^15^ (Supplemental Fig. 9). Furthermore, our theoretical model based solely on steric considerations was able to accurately reproduce the product distributions in the mutants considered.

The identified active site clamp not only fixes the pentadiene system of the substrate, but most likely shields C9 and C13 in case of linoleic acid from dioxygen, perhaps in combination with other steric effects (Fig. 3A). This suggests that the substrate is very tightly bound in the active site clamp and a reaction in the thermodynamically favored way is hindered. Steric hindrance also seems to control the specificity of MnLOX. Here, two pockets were identified in the active site that differ in size in Mo-MnLOX and Gg-MnLOX. It was therefore concluded that the width of the substrate channel and the two side pockets may specifically control oxidation at C-9 or C-13 of linoleic acid by Mo-MnLOX and Gg-Mn-LOX^14^. In addition to the residues identified in CspLOX2, Phe337 and Phe332 of Gg-MnLOX and Mo-MnLOX, respectively, could have a similar shielding function^14, 15^ (Supplemental Fig. 9).

Localization of spin density at C-11 would provide an elegant explanation for the bis-allylic product. In fact, a correlation between calculated spin densities and product distributions suggested that dioxygen is preferentially added to the carbon atom with the highest spin density of a delocalized radical^24, 28^. However, it seems very unlikely that this could play an important role for the specificity of CspLOX2, as indicated by our model DFT calculations (Fig. 5), and the dihedral distributions extracted from the MD simulations. Similar results were obtained for the cyclooxygenase enzyme that catalyzes dioxygen addition to C-11 of arachidonic acid to form a conjugated product^23^.

The question, how molecular oxygen is delivered to the substrate in the active site channel is not yet unambiguously answered. Although it is possible that dioxygen enters through the same channel as the substrate, an additional oxygen transport channel that enters the active site opposite of the iron has been postulated in several LOX, including sLOX1^27, 29^, rabbit 12/15-LOX^30^, human 15-LOX2^31^, porcine 12-LOX^32^ and coral 8*R*-LOX^31^. We also found a putative channel in CspLOX2 at the same position that might serve as oxygen channel as well (Supplemental Fig. 10). This putative channel connects the surface of the protein with the base of the substrate-binding channel at the opposite site of the iron, which is in line with the antarafacial oxidation of the substrate and a high dioxygen concentration at the pro-*R* face of C-11. In addition, the model of the Gly/Ala switch matches with this oxygen channel too, since it is located at the connection of the putative oxygen channel to the substrate channel to efficiently control the access of dioxygen. Such an oxygen channel may also explain why dioxygen is preferentially delivered to 9 *S* and 13 *R* of linoleic acid and why the results of the MD simulations correlate nicely with the mutations on the opposite side of the iron (Fig. 6). However, further evidence will be required to confirm the function of this channel.

To be sure about the localization of the substrate in the active site, a crystal structure of the bound substrate will be required. It is feasible that the active site clamp has a similar importance in other LOX and that in addition to the depth of the binding and the orientation of the substrate, the dioxygen access to 9*R*, 9*S*, 13*R* and 13*S* regulated by surrounding amino acids is just as important for the LOX specificity.

## Materials and Methods

### Media and Reagents

Chemicals were obtained from Sigma and Carl Roth & Co. Agarose was from Biozym Scientific GmbH. All fatty acids were from Sigma or Cayman Chemical. Acetonitrile was from Fisher Scientific. Restriction enzymes were purchased from MBI Fermentas.

### Recombinant Protein Expression and Purification

Site-directed mutagenesis of the pET28a based constructs^12, 22^ was performed according to the QuickChange II Site-Directed Mutagenesis kit (Agilent Technologies) and the correctness of the resulting plasmids was confirmed by sequencing. For heterologous expression, His-tagged recombinant proteins were produced in *E. coli* BL21 Star cells (Invitrogen). For high yields of the recombinant protein, cells were grown in the auto-induction medium ZYP-5052 supplemented with 25 µg/ml kanamycin and 250 µM ammonium iron (III) citrate^33^. The expression cultures were first incubated at 37 °C for 2.5 h and then further cultivated at 28 °C for 24 h while shaking. Cells were harvested at 2500 g at 4 °C for 20 min, frozen in liquid nitrogen and stored at −20 °C. For purification of CspLOX1 and CspLOX2, the cell pellet was resuspended in buffer A (50 mM Tris/HCl pH 8, 100 mM NaCl) and 1 mg/ml lysozyme, 0.2 mM phenylmethylsulfonyl fluoride and 20 µg/ml DNase were added. After incubation on ice for 30 min, the cells were disrupted using a fluidizer. The cell lysate was separated from the cell debris by centrifugation at 50000 g at 4 °C for 20 min and the supernatant was applied to a preequilibrated 5 ml His-Trap FF column (GE Healthcare). Unspecifically bound proteins were removed by washing with 50 mM Tris/HCl pH8, 100 mM NaCl, 15 mM imidazole. Finally, the protein was eluted with 50 mM Tris/HCl pH 8, 100 mM NaCl, 500 mM imidazole. For crystallization, the proteins were further purified by size exclusion chromatography using a Superdex 200 HR (GE Healthcare) equilibrated with buffer A and concentrated to 5–10 mg/ml in a Spin-X UF concentrator (cutoff of 30,000, Corning).

### Enzyme activity assay and analysis of products

The enzymatic activity of CspLOX2 and its variants was assayed measuring the decrease in oxygen concentration using an oxygen electrode. For measuring v_max_ under substrate saturation conditions, 15 µg enzyme was added to 1 ml of 100 µM LA in 200 mM sodium borate buffer. For analysis of the enzymatic activity of CspLOX1, the formation of conjugated double bonds was alternatively monitored at 234 nm (ε = 2.5 × 10^4^ M^−1^ cm^−1^) with a CARY 100 Bio spectrophotometer (Varian). For the HPLC analysis of reaction products, the reaction was quenched directly when the first steep increase at 234 nm was completed, which corresponds to the time point, when highest concentrations of 11-HPODE are reached^13^. The reaction was stopped by mixing the content of the cuvette with 1 ml diethylether and cooling it on ice. The hydroperoxide products were quickly extracted with diethylether and directly separated by SP-HPLC on a Zorbax Rx-SIL column (150 × 2.1 mm, 5 µm particle size, Agilent) with a solvent system of n-hexane/2-propanol/acetic acid (100:1:0.1, v/v/v) at a flow rate of 0.2 ml/min. Absorbance at 234 nm was recorded for the detection of conjugated hydroperoxy fatty acids, while the absorbance at 202 nm was used for the detection of 11-HPODE^12^. For the separation of the stereo isomers of each product, the hydroperoxides were reduced to the corresponding hydroxides with sodium borohydride prior to extraction with diethylether. The fatty acid hydroxides were separated on a Chiralcel OD-H column (150 × 2.1 mm, 5 µm particle size, Daicel, VWR) with a solvent system of n-hexane/2-propanol/trifluoroacetic acid (100:5:0.1, v/v/v) at a flow rate of 0.1 ml/min.

### Separation of 11*R*- and 11*S*-HODE

For CP-HPLC analysis of 11-HPODE, the products were derivatized to the hydroxy octadecadienoic acid methyl esters (HODE-Me). For reduction, sodium borohydride was added before extraction with diethylether. The extracted fatty acid hydroxides were methylated by addition of 400 methanol and 6 µl diazomethane. After 30 min, the reaction was stopped with 2.5 µl 1:10 (v/v) acetic acid. 11-HPODE-Me was separated from the conjugated products by SP-HPLC. 11-HODE-Me was collected, dried and subjected to CP-HPLC analysis at a flow rate of 0.1 ml/min with a solvent system of *n*-hexane/2-propanol/acetic acid (100:0.3:0.05).

### Crystallization

Crystals of the wt protein were grown by the sitting drop vapor diffusion method in a well solution of 10% polyethylene glycol 4000, 0.1 M MES/imidazole pH 6.5, 20% glycerol and 0.02% alcohols (1,6-hexanediol, 1-butanol, 1,2-propanediol, 2-propanol, 1,4-butanediol, 1,3-propanediol) and grew to a size of 70 × 20 × 30 µm within 10 days. Crystals were mounted on a loop and immediately flash-cooled in liquid nitrogen. Crystals of CspLOX2 variants were grown as a hanging drop in a well solution of 12.5–15 polyethylene glycol 4000, 8–12% glycerol, 0.1 M MES/imidazole pH 6.1, and 0–600 mM NaCl. For these crystals, a solution of 20% glycerol, 15% polyethylenglycol 4000 and 0.1 M MES/imidazol pH 6.1 was used as a cryoprotectant.

### Data collection and processing

Diffraction data to 1.8 Å resolution were recorded from a single crystal on beamline BL-14.1 at BESSY II (Berlin, Germany)^34^ equipped with MAR CCD detector (MOSAIC 225) and processed and scaled with the XDS package^35^. The oscillation images could be processed in either space group P222 or P422 with almost identical results (Rmerge = 7.1% in P222, 7.1% in P422) due to very similar lengths of two unit cell parameters. The hypothesis that the crystal was twinned was tested by a statistical analysis using phenix. xtriage^36^. The results of the L-test indicated that no twinning was suspected. The Stanley factor 〈I^2^〉/〈I〉^2, 37, 38^ was found to be 1.865 (standard values of the 〈I^2^〉/〈I〉^2^ ratio are ∼2.0 for untwinned data and ∼1.5 for twinned data). Statistics for the data in P222 are presented in Table 2.

**Table 2:** Data collection and refinement statistics.

**Data collection**	CspLOX2 wt	CspLOX2 L304V	CspLOX2 L304F	Mn-CspLOX2
Space group	P2_1_2_1_2_1_	P2_1_2_1_2_1_	P2_1_2_1_2_1_	P2_1_2_1_2_1_
**Cell dimensions**				
a, b, c (Å)	54.50, 165.40, 166.21	54.71, 167.10, 167.16	54.40, 166.26, 166.74	54.42, 165.59, 167.35
X-ray source	BL 14.1 (BESSY Berlin)	ID23-2 (ESRF Grenoble)	ID23-2 (ESRF Grenoble)	P14 (PETRA III, Hamburg)
Resolution range (Å)	45.0–1.80 (1.90–1.80)	46.35–2.50 (2.60–2.50)	46.21–2.36 (2.46–2.36)	46.26–2.00 (2.10–2.00)
No. of unique reflections	134076	53895	62963	102444
Completeness (%)	95.7 (76.7)	99.7 (99.9)	99.2 (99.5)	99.7 (99.9)
R_merge_ (%)	7.1 (54.2)	4.8 (60.0)	4.1 (53.3)	7.0 (79.1)
Average I/σ	10.1 (2.1)	22.7 (3.0)	18.5 (2.7)	15.0 (1.7)
Redundancy	3.2 (2.3)	5.7 (6.0)	3.8 (3.8)	4.4 (4.3)
CC_1/2_	99.6 (71.2)	99.9 (85.9)	99.9 (80.7)	99.3 (99.2)
Wilson B-factor (Å^2^)	24.5	65.1	52.3	32.3
**Refinement**				
Resolution (Å)	41.55–1.8 (1.82–1.80)	46.35–2.50 (2.55–2.50)	46.21–2.36 (2.39–2.36)	46.26–2.00 (2.05–2.00)
No. of reflections	133973	48349	62942	102398
R_work_ (%)	17.35 (28.12)	19.26 (28.42)	19.07 (28.07)	18.18 (31.78)
R_free_ (%)	20.29 (35.50)	22.00 (35.80)	21.62 (28.57)	20.51 (34.13)
Total number of atoms/average B-factor (Å^2^)	10296/30.3	9260/67.0	9503/59.6	10169/39.4
Protein residues/average B-factor (Å^2^)	1134/29.2	1134/67.0	1135/59.7	1132/38.8
Water molecules/average B-factor (Å^2^)	1051/38.2	114/54.9	364/53.5	1049/44.5
Ligand molecules, ions/average B-factor (Å^2^)	19/44.5	14/86.2	14/75.0	9/47.1
Bond lengths (Å)	0.008	0.007	0.008	0.003
Bond angles (°)	1.044	0.761	0.707	0.590
**Ramachandran statistics**				
favored (%)	97.43	97.61	97.79	97.69
allowed (%)	2.57	2.39	2.13	2.31
outliers (%)	0.00	0.00	0.09	0.00
PDB accession code	5MED	5MEE	5MEF	5MEG

### Structure solution and refinement

The crystal structure of holo CspLOX2 was solved by Molecular Replacement method (MR) with PHASER program^39^ using 7 highest scored templates identified by HHPRED server^40^ as the search models (PDB ids: 1f8n, 1lox, 2iuj, 2iuk, 3dy5, 3fg1, 3o8y). Initial attempts to solve the structure by MR were carried out in the tetragonal space group P4_3_2_1_2 with one molecule occupying the asymmetric unit. The best fitting model was selected based on crystallographic R factors upon refinement of seven individual MR solutions in PHENIX. The model with lowest R factors derived from *homo sapiens* 5-lipoxygenase (PDB id: 3o8y) was remodeled by combining homology modeling with density-guided energy optimization as implemented in the Rosetta package^41^ and extensively refined in PHENIX and remodeled manually in Coot^42^. The best model in P4_3_2_1_2 space group could be refined to R = 36.2% and Rfree = 41.1%.

The failure to refine the structure successfully in a tetragonal system indicated over-merging of diffraction data and implicated orthorhombic symmetry of the crystal. Molecular Replacement was carried in PHASER against orthorhombic diffraction data using the search model derived from *homo sapiens* 5-lipoxygenase (PDB id: 3o8y) and resulted in placing two CspLOX2 molecules in the asymmetric unit. Those two molecules are related by a rotation of 179.8 degrees around an axis mimicking a diagonal 2-fold axis in the tetragonal system, what explained the observed pseudo tetragonal symmetry. The initial model was remodeled with Rosetta^41^ using the calculated electron density map as restraints and further manually rebuild and refined in Coot and PHENIX, respectively. The final model consisting of residues 1 to 569, two Fe(II) ions, 1069 water molecules, 3 1-butanol molecules, 6 1,2-propanediol molecules and 1 glycerol molecule was refined at 1.8 Å resolution to crystallographic R and Rfree factors of 16.14% and 19.50%, respectively. The two CspLOX2 molecules exhibit high structural similarity as judged based on calculated root mean square deviation (RMSD) of 0.25 Å calculated for 565 compared Cα atoms. The stereo chemical quality of the structure was validated using PHENIX implementation of MolProbity^43^. The crystal structures of L304V and L304F variants and Mn-CspLOX2 were solved using the Difference Fourier Method using the protein part of holo CspLOX2 structure as the starting model. All structures were refined and manually rebuild in PHENIX and Coot, respectively. Data collection, structure refinement statistics and validation are summarized in Table 2.

### Computational details

Calculations on the 2,5-heptadienyl radical model system were performed at the B3LYP-D3/def2-TZVP level of theory. The spin density distributions of the SOMO were obtained using Molpro2012.1^44^. The activation barriers were extracted from relaxed surface scans with fixed C9-C10-C11-C12 and C10-C11-C12-C13 dihedrals approaching oxygen in steps of 0.1 Å using the ORCA quantum chemistry package^45^.

In order to perform MD simulations the chain A of the CspLOX2 crystal structure was saturated with hydrogens and placed in a water box with 8 Å distance to the periodic cell limits. The charge in the cell was neutralized by adding 11 Na^+^ ions. 2000 steps of minimization were carried out placing a restraint of 5 kcal/Å^2^ on all non-H atoms in the enzyme, followed by 3000 steps with no restraints. All atoms were modeled with the Amber ff10 force field^46^. A non-bonded sphere model was used for the Fe^2+^ and the parameters for the radical substrate were taken from Furse *et al*.^23^ using Merz-Kollman charges at the B3LYP/6-31 G* level of theory. Single amino acid mutations were generated by replacing the corresponding residue manually. For each enzyme, three starting structures were prepared by placing the radical of linoleic acid in the active site pocket in different conformations. The SHAKE algorithm was used for all dynamic runs, with a 2 fs time step, and a 12 Å cutoff for non-bonded interactions. A Langevin thermostat was used throughout. After 50 ps of heating to 300 K, 350 ps of NPT dynamics were carried out at ambient conditions for equilibration. The production runs consisted of 1 ns for each substrate conformation taking 5000 snapshots for steric shielding analysis.

### Data availability

Coordinates and structure factors have been deposited within the Protein Data Bank (PDB codes 5MED, 5MEE, 5MEF and 5MEG). All other datasets generated during the current study are available from the corresponding author on reasonable request.

## Electronic supplementary material

Supplementary Figures
