# Supplementary material for: Lipoxygenase 2 from Cyanothece sp. controls dioxygen insertion by steric shielding and substrate fixation
Source: Sci Rep. 2017 May 18;7:2069. doi: 10.1038/s41598-017-02153-w (PMC5437038; doi:10.1038/s41598-017-02153-w)
Supplement: Supplementary file 1 — Supplementary Figures [file 41598_2017_2153_MOESM1_ESM.pdf]

Supplementary Information

Lipoxygenase 2 from Cyanothece sp. controls dioxygen insertion by steric shielding and substrate fixation

by Julia Newie, Piotr Neumann, Martin Werner, Ricardo A. Mata, Ralf Ficner and Ivo Feussner

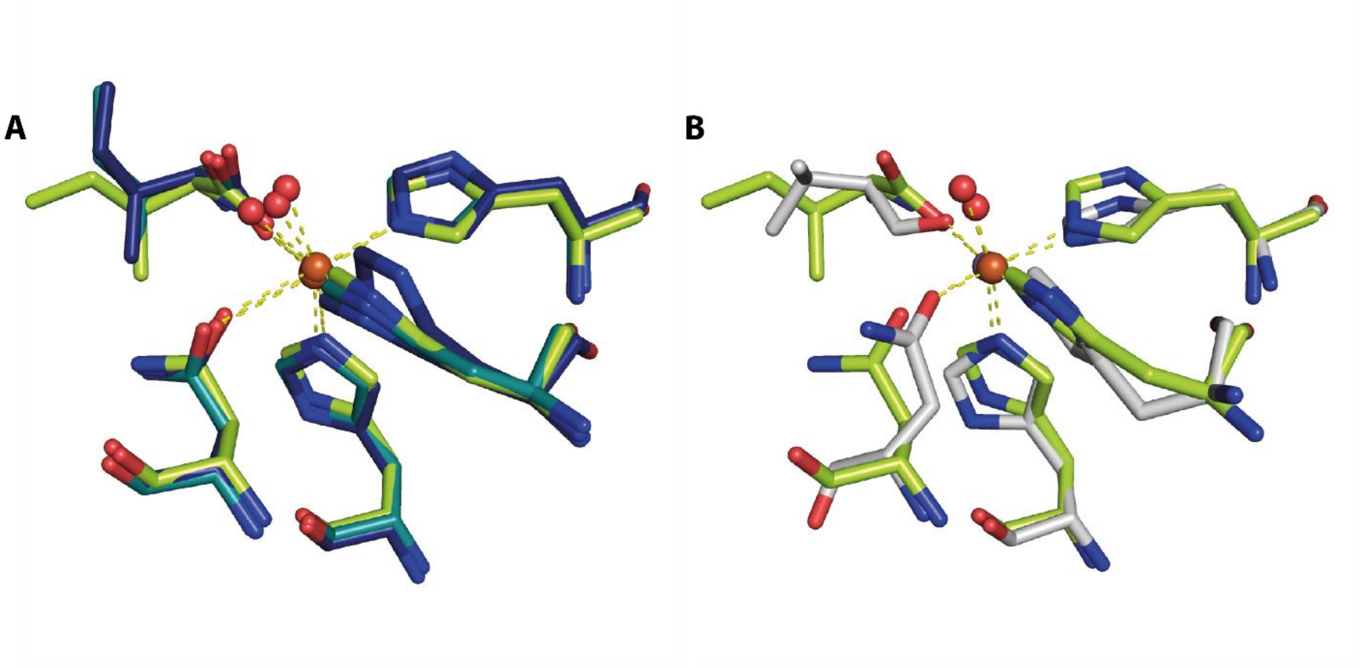

Supplemental Figure 1: Coordination sphere of CspLOX2 in comparison with iron and manganese containing LOX. A. Comparison with FeLOX. CspLOX2 (PDB code 5MED) is shown in light green, coral 8*R*-LOX (PDB code 4QWT) in dark green and soybean sLOX1 (PDB code 3PWZ) in dark blue. Iron is depicted as orange sphere and the coordinated water as red sphere. B. CspLOX2 in comparison with Gg-MnLOX (grey, PDB code 5FX8).

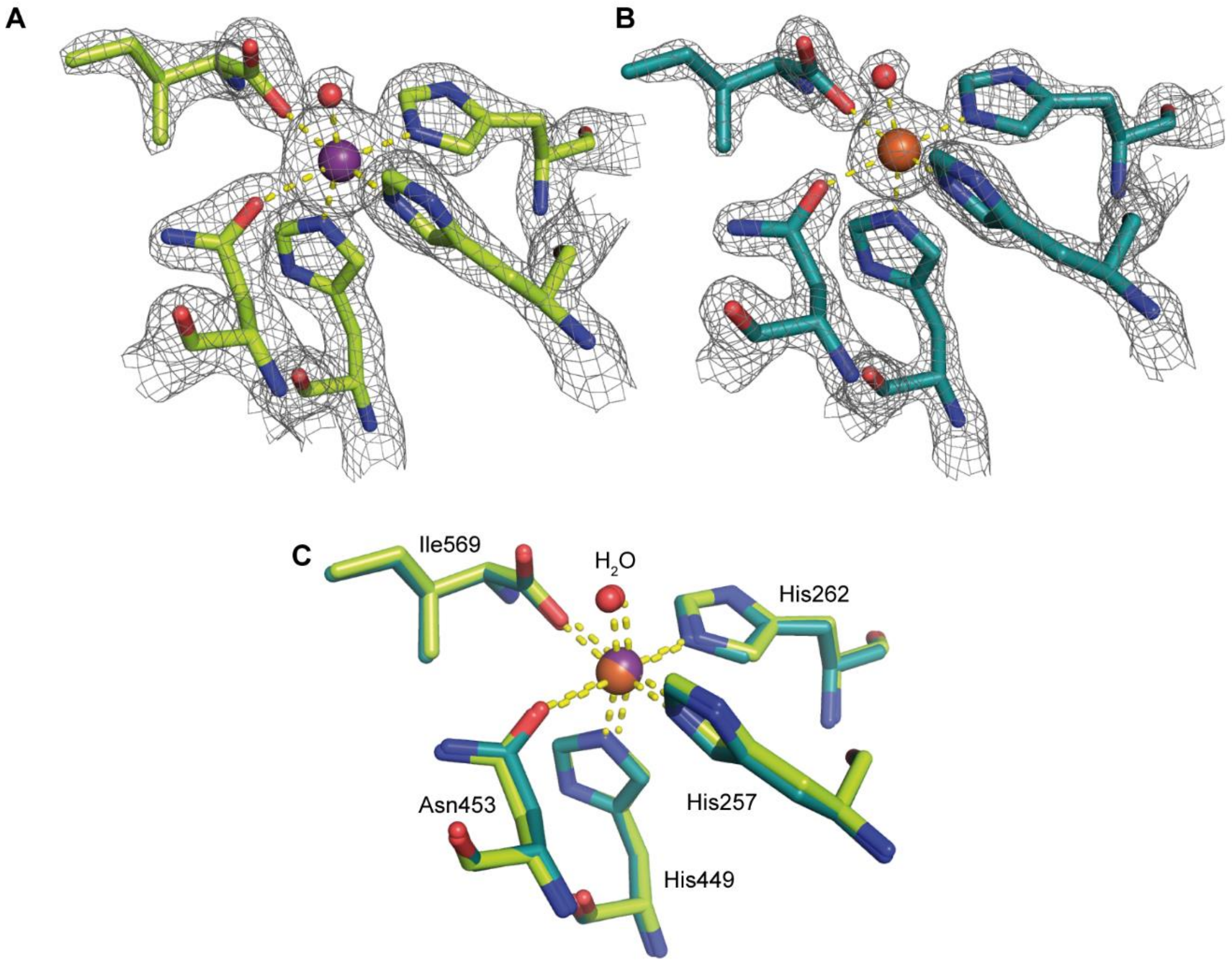

Supplemental Figure 2: Comparison of the coordination spheres of (A) Mn-CspLOX2 (PDB code 5MEG) and (B) Fe-CspLOX2 (PDB code 5MED). The 2mFo-DFc electron density maps were contoured at 2.8 σ for Mn-CspLOX2 and 4 σ for Fe-CspLOX2, respectively. (C) Superposition of both Mn-CspLOX2 and Fe-CspLOX2 coordination spheres reveals that those are virtually identical. Mn-CspLOX2 is shown in light green with Mn as purple sphere and Fe-CspLOX2 is shown in dark green with Fe as orange sphere.

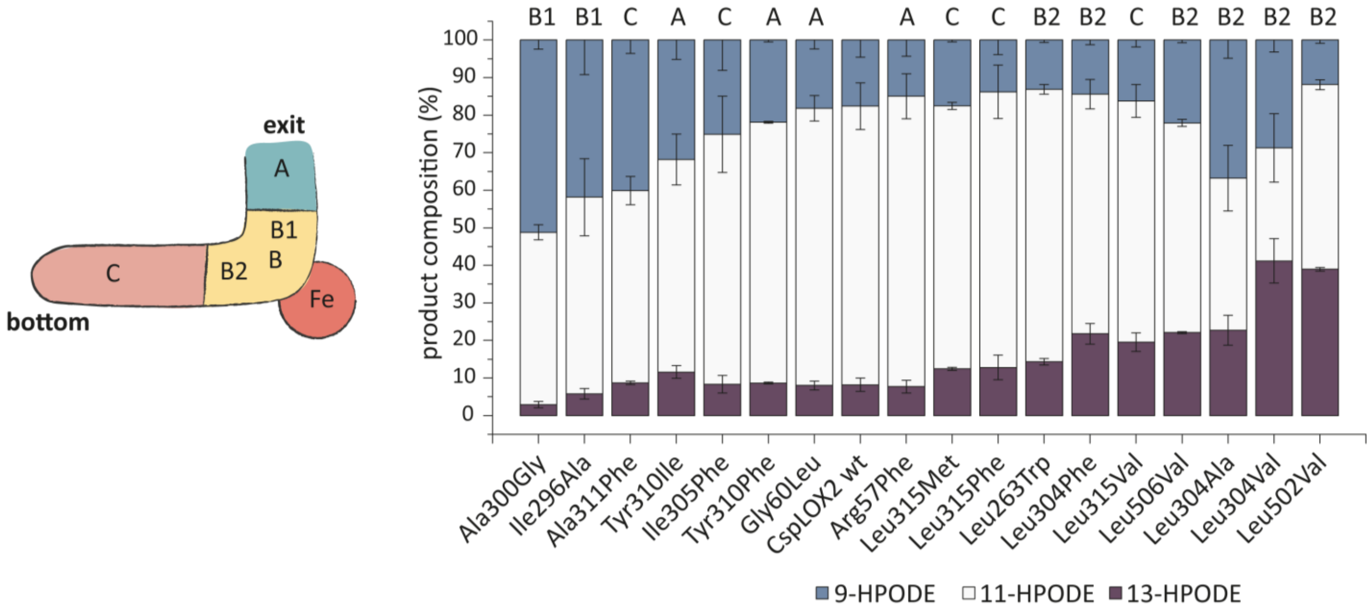

Supplemental Figure 3. Mutations throughout the active site channel affect the regio and stereospecificity of CspLOX2. (Left) Schematic representation of the putative substrate-binding channel. (Right) The residues are categorized according to the areas shown right. The product distribution of the generated mutants was analyzed by HPLC. Shown are mean values and error bars show standard deviation. All experiments were carried out in triplicates.

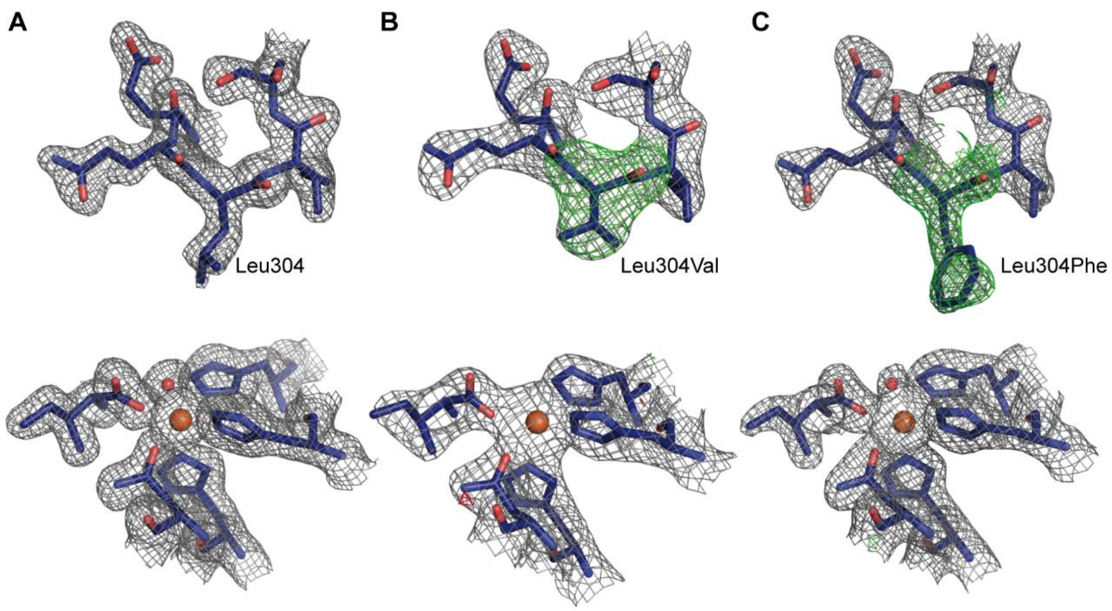

Supplemental Figure 4: Structures of (A) wt CspLOX2 (PDB code 5MED) compared with the variants (B) Leu304Val (PDB code 5MEE) and (C) Leu304Phe (PDB code 5MEF). Only the exchanged amino acid is affected by the mutation. The rest of the protein remains unchanged (calculated RMSD on 1134 Ca atoms amounts to 0.25 Å and 0.26 Å for wt/Leu304Val and wt/ Leu304Phe pair, respectively). The *2mFoDFc* electron density maps are contoured at 1.5 σ and the omit maps, shown in green (*mFo-DFc*, excluding the residue at position 304), are contoured at 4 σ. The catalytic iron is shown as orange sphere and the coordinated water/hydroxide as small red sphere.

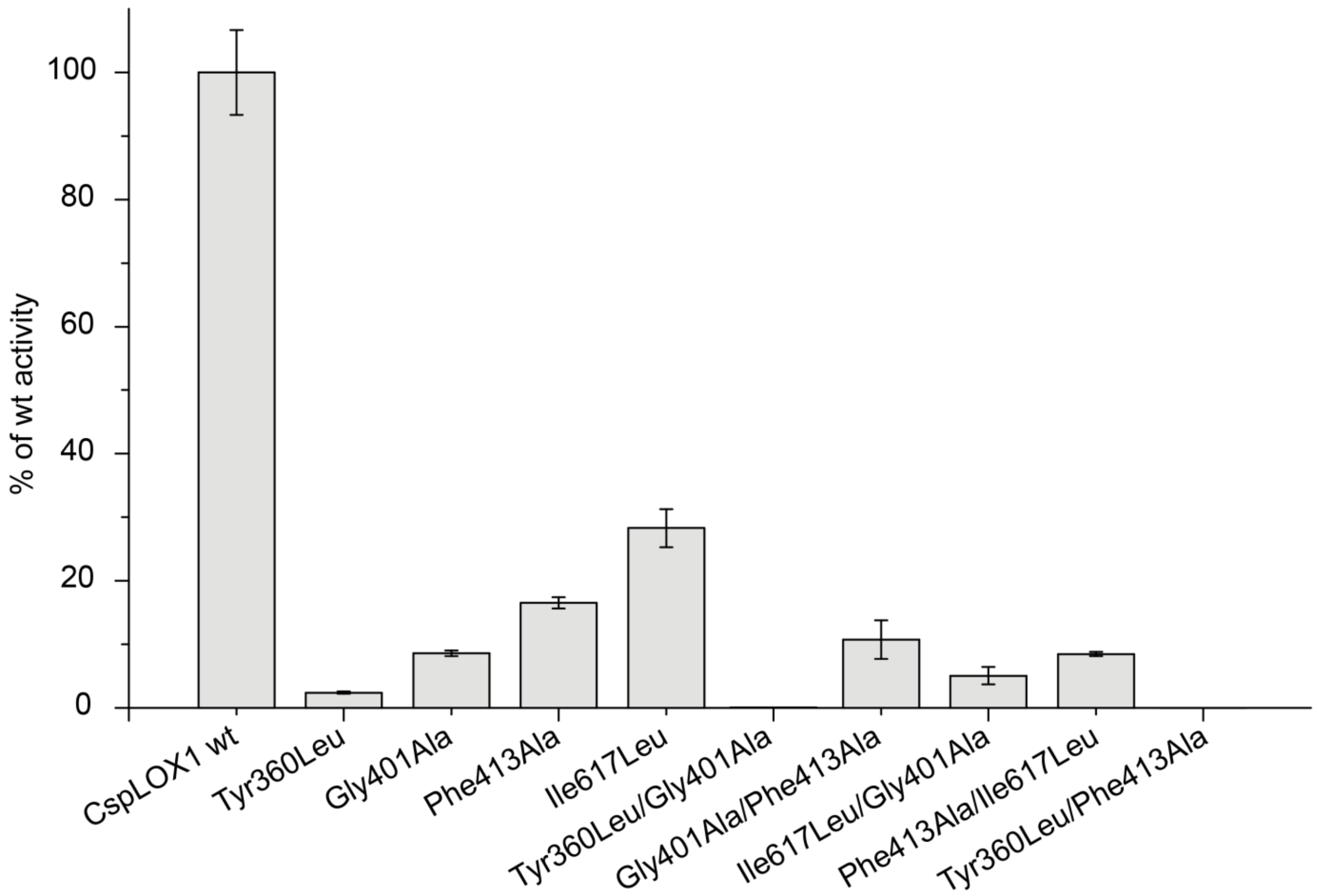

Supplemental Figure 5: Catalytic activity of CspLOX1 and its variants. Proteins were purified by IMAC. The relative activity was determined at 234 nm using a spectrophotometer. Triple mutants were inactive and are not shown in this figure. Depicted are the mean values and standard deviations of three experiments.

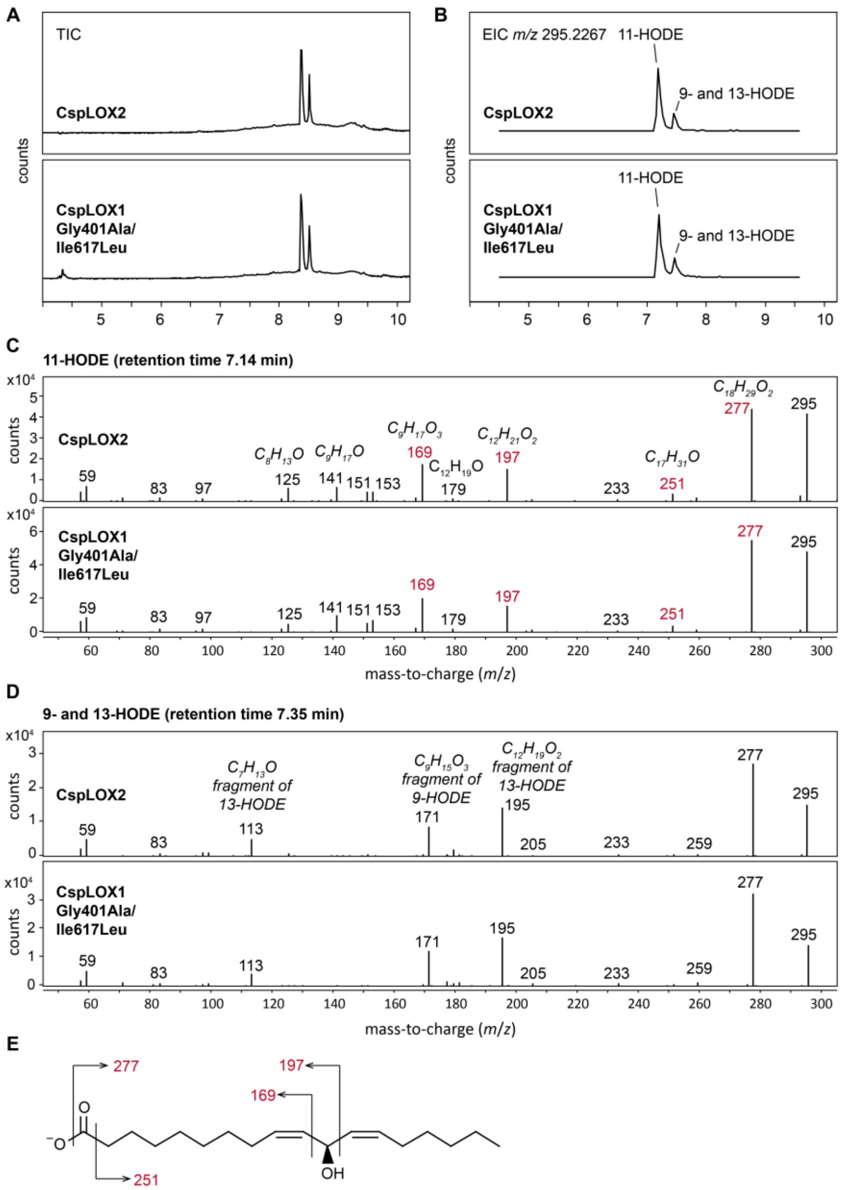

Supplemental Figure 6: Confirmation of the 11-HPODE species produced by the Gly401Ala/Ile617Leu variant of CspLOX1. 11-HPODE produced by CspLOX2 and the CspLOX1 variant were reduced to hydroxides, purified by HPLC and subjected to UHPLC-QTOF MS. (A) The total ion chromatogram (TIC) is shown for both samples analyzed in the negative ionization mode and (B) the corresponding extracted ion chromatogram (EIC) at *m/z* 295.2267. (C) The high resolution mass spectrum (resolution < 2ppm) for the first peak eluting at 7.14 min corresponds to 11-HODE, while (D) the mass spectrum for the peak eluting at 7.35 min corresponds to 9- and 13-HODE. The exact mass information of the MS/MS fragments was used for deducing the elemental composition. (E) Fragmentation pattern of 11-HODE and the resulting *m/z* signals.

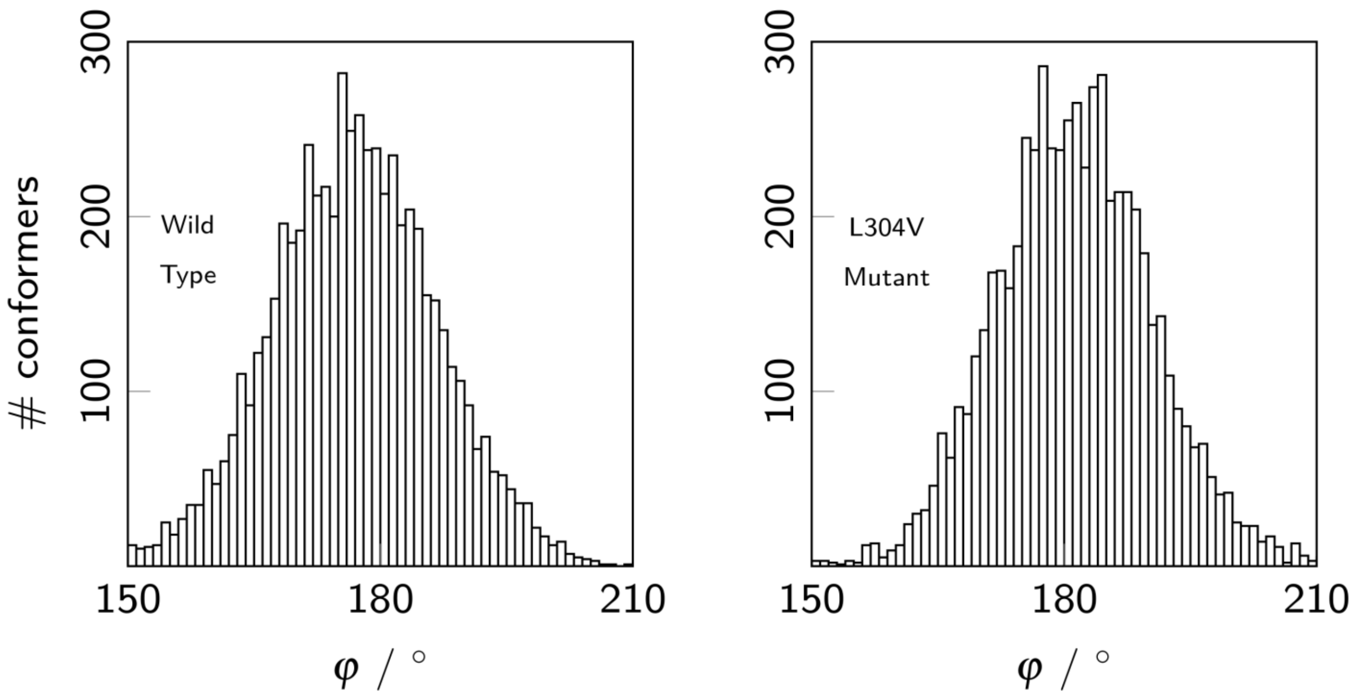

Supplemental Figure 7: Dihedral angle distributions in CspLOX2 wild type and the Leu304Val mutant. The distribution of an internal substrate dihedral angle extracted from MD simulations. Both histograms exhibit a maximum close to the preferred planar substrate conformation.

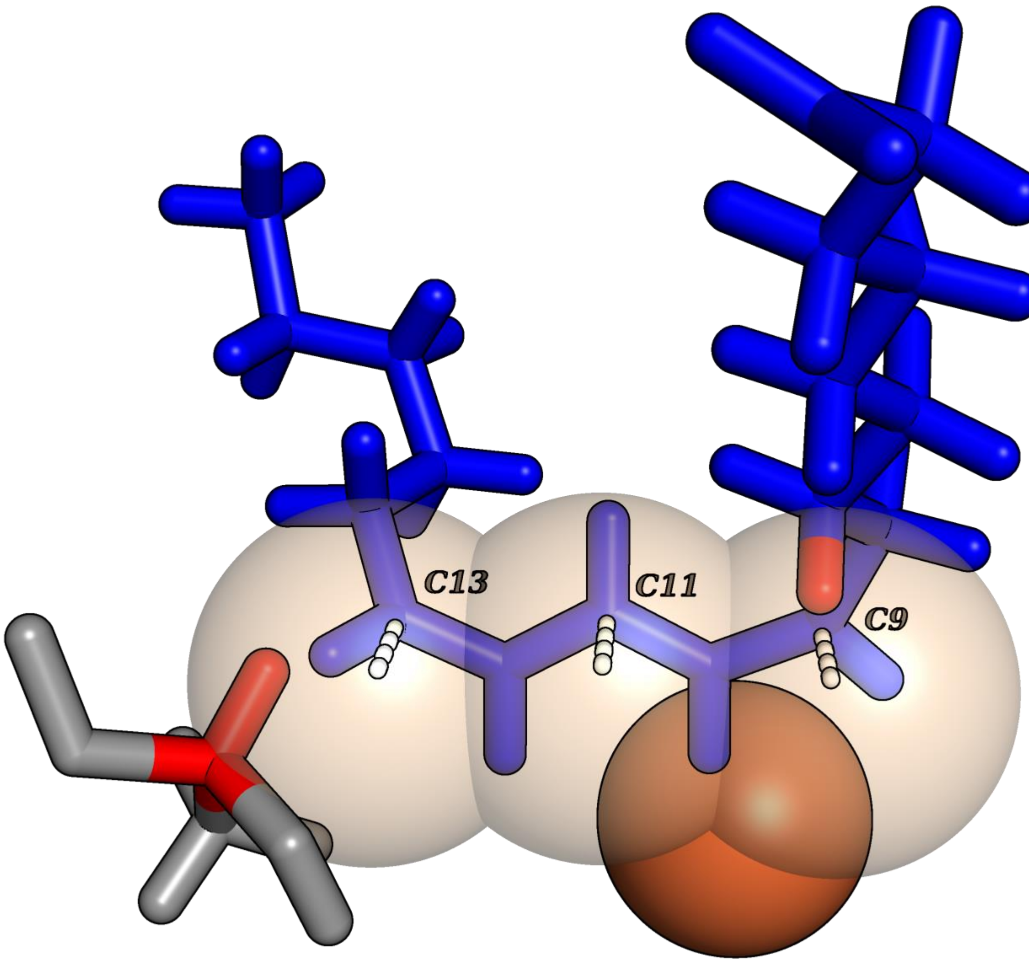

Supplemental Figure 8: An equalization plane is defined through the substrate (blue) and points are found in 3 Å distance along the normal of this plane as indicated by the dotted lines. If atoms (red) are found within a certain sphere around that point the corresponding site is considered to be less accessible. In the case shown, C9 would be partly shielded by a substrate atom while C13 accessibility is reduced by an amino acid residue. C11 would be the only site to be considered fully accessible. The iron of the active site is shown in the background.

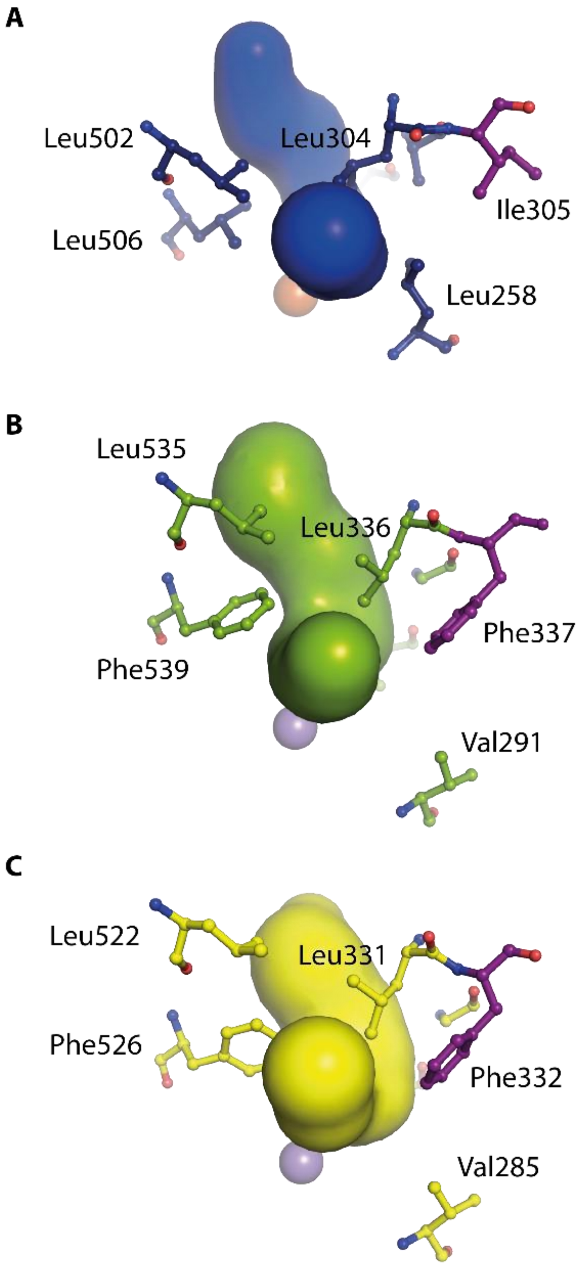

Supplemental Figure 9: Comparison of the active site clamp of CspLOX2 (A) with those of Gg-MnLOX (B) and Mo-MnLOX (C). The channels were computed with Caver 3.0 and are shown with the bottom in front and with the Fe (orange sphere) or Mn (purple sphere) cofactor at the base. While Phe337 and Phe332 are lining the active sites of Gg-MnLOX (PDB code 5FX8) and Mo-MnLOX (PDB code 5FNO), respectively, the corresponding residue in CspLOX2 (Ile305) is pointing away from the putative substrate channel (shown as purple sticks).

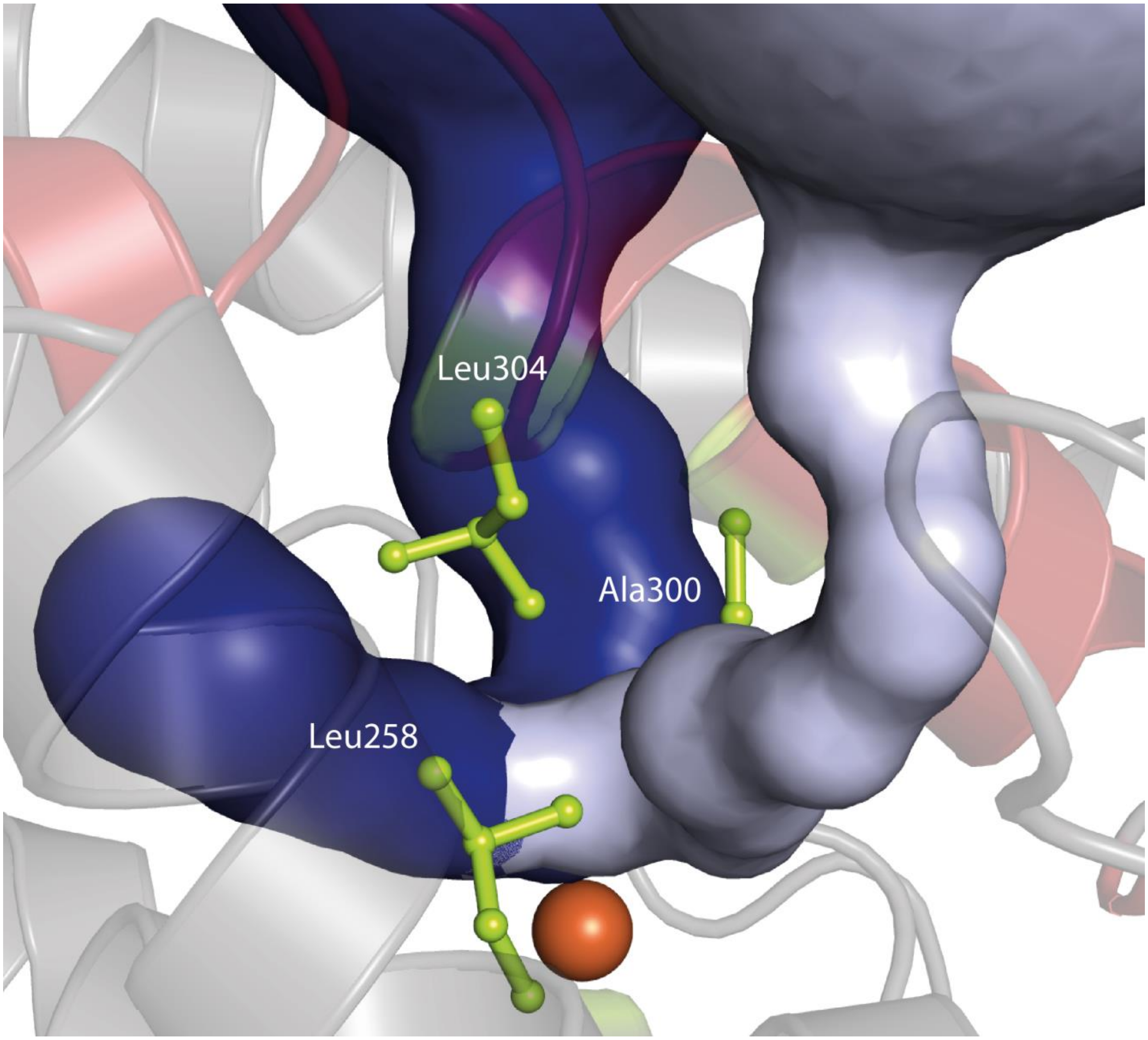

Supplemental Figure 10: Putative oxygen channel of CspLOX2. The channels were calculated with Caver 3.0. The substrate binding channel is shown in dark blue, while the putative oxygen binding channel is shown in light blue. Both channels are separated by the so-called “arched helix” (red). The connection of both channels is positioned close to the Fe cofactor (orange sphere) and is surrounded by Leu258, Ala300 and Leu304 (green).
